# Genome-wide analysis of the *Tritipyrum* NAC gene family and the response of *TtNAC477* in salt tolerance

**DOI:** 10.1186/s12870-023-04629-6

**Published:** 2024-01-09

**Authors:** Xiaojuan Liu, Guangyi Zhou, Songshu Chen, Zhenzhen Jia, Suqin Zhang, Fang He, Mingjian Ren

**Affiliations:** 1 Guizhou Subcenter of National Wheat Improvement Center, Key Laboratory of Molecular Breeding for Grain and Oil Crops in Guizhou Province, Agronomy College, Guizhou University, Guiyang, 550025 China; 2 School of Life Sciences, Guizhou Normal University, Guiyang, 550025 China

**Keywords:** *Tritipyrum*, WRKY, Salt-tolerance, *TtNAC477*, Genome-wide, Expression patterns

## Abstract

NAC transcription factors are widely distributed in the plant kingdom and play an important role in the response to various abiotic stresses in plant species. *Tritipyrum*, an octoploid derived from hybridization of *Triticum aestivum* (AABBDD) and *Thinopyrum elongatum* (EE), is an important genetic resource for integrating the desirable traits of *Th. elongatum* into wheat. In this study, we investigated the tissue distribution and expression of *Tritipyrum* NAC genes in the whole genomes of *T. aestivum* and *Th. elongatum* after obtaining their complete genome sequences. Based on phylogenetic relationships, conserved motifs, gene synthesis, evolutionary analysis, and expression patterns, we identified and characterized 732 *Tritipyrum* NAC genes. These genes were divided into six main groups (A, B, C, D, E, and G) based on phylogenetic relationships and evolutionary studies, with members of these groups sharing the same motif composition. The 732 *TtNAC* genes are widely distributed across 28 chromosomes and include 110 duplicated genes. Gene synthesis analysis indicated that the NAC gene family may have a common ancestor. Transcriptome data and quantitative polymerase chain reaction (qPCR) expression profiles showed 68 *TtNAC* genes to be highly expressed in response to various salt stress and recovery treatments. Tel3E01T644900 (*TtNAC477*) was particularly sensitive to salt stress and belongs to the same clade as the salt tolerance genes *ANAC019* and *ANAC055* in *Arabidopsis*. Pearson correlation analysis identified 751 genes that correlated positively with expression of *TtNAC477*, and these genes are enriched in metabolic activities, cellular processes, stimulus responses, and biological regulation. *TtNAC477* was found to be highly expressed in roots, stems, and leaves in response to salt stress, as confirmed by real-time PCR. These findings suggest that *TtNAC477* is associated with salt tolerance in plants and might serve as a valuable exogenous gene for enhancing salt tolerance in wheat.

**Supplementary Information:**

The online version contains supplementary material available at 10.1186/s12870-023-04629-6.

## Introduction

Salinization of the soil is among the most consequential types of abiotic stress. Globally, salt stress impacts an estimated 8.31 × 10^8^ hectares of soil and results in annual economic losses of $27.3 billion as a consequence of reduced agricultural productivity [1, 2]. Grain production in China's highly productive farmland has decelerated in recent years, while expansion potential remains in medium- to low-yield farmland. It has become an urgent matter to determine how to optimally utilize these marginal farmlands and salinity-affected regions in order to increase overall grain production. Salt stress can give rise to a multitude of secondary stresses, ion toxicity and osmotic stress [3]. Cell growth and metabolic activity can be adversely affected by the accumulation of diverse stress conditions, which in turn can have an impact on seed germination, seedling development, and yield [4]. To enhance their chances of survival, plants undergo various morphological, physiological, and molecular modifications. These include reduction in leaf number and size, stomatal closure, efflux and compartmentalization of Na^+^ and Cl^−^ ions, scavenging of reactive oxygen species, accumulation of osmoprotectants, and expression of genes that are responsive to stress [5, 6]. The regulation of physiological metabolism levels and the improvement of morphological structures is influenced by the expression of stress-responsive genes, in which transcription factors play a pivotal role [7].

After perception of a stress signal, plant cells transmit the signal to stress-responsive transcription factors (TFs) through specific signalling pathways. These TFs have the ability to bind to cis-acting elements in the upstream promoter region of target genes via their DNA-binding domains (DBDs) to regulate expression of target genes in diverse plant tissues, cells, and environmental conditions. Such activation of the stress response in plants helps to mitigate the damage caused by stress [8, 9]. NAC TFs, named for initials NAM (from *Petunia hybrida*), *ATAF1*/2, and CUC2 (from *Arabidopsis thaliana*), comprise one of the largest TF families in plants [9]. A prominent characteristic of NAC TFs is the highly conserved NAC structural domain (150–160 amino acids) at the N-terminus of the protein; the C-terminus consists of a variable transcriptional regulatory region (TRR). The NAC domain serves as the binding domain for NAC TFs and is subdivided into five substructural domains (A-H). Among these, the C and D substructural domains are significantly conserved and contain nuclear localization signals that are also associated with DNA binding. In contrast, the B and E substructural domains exhibit variability and may confer distinct functions to NAC members [10]. In addition to the NAC structural domains, numerous members within the same group share one or more motifs. For example, Nuruzzaman et al. demonstrated that the TRR motif is conserved within specific NAC subfamilies but varies between subfamilies, thus providing individual functional variations to NAC proteins [11]. Some NAC TFs also possess transmembrane (TM) motifs in their C-terminal region, which facilitate localization to the plasma membrane or endoplasmic reticulum. These proteins are cleaved through proteolytic hydrolysis and subsequently translocated to the nucleus under specific developmental stages or stress conditions [12]. Notably, analysis of the tertiary structure of plant NAC proteins is limited, and only the conserved structural domains of 2 NAC TFs, *ANAC019* and *OsNAC*, have been determined via X-ray diffraction [13]. *ANAC019*, the first NAC protein with a resolved crystal structure, exhibits an N-terminal conserved structural domain composed of three α-helices and six antiparallel β-folds. The homodimerization model of *ANAC019* has also been reported, suggesting that many NAC proteins exert their regulatory effects as homodimers due to the presence of similar dimer-binding sites [14].

The structural diversity of NAC proteins enables them to perform multiple functions, thus making NAC TFs crucial in various stages of plant growth and development and under different stress conditions [15]. In tomato, expression of *SlNAC35* is triggered by drought stress, salt stress, and bacterial pathogens. Overexpression of *SlNAC35* in transgenic tobacco enhances resistance to drought and salt stress and confers tolerance to bacterial pathogens [16]. In *Arabidopsis*, the role of *AtAF1* is to negatively regulate plant defence responses to grey mould. On the other hand, *AtAF2* is induced at high levels at the site of leaf injury and in response to injury-associated methyl jasmonate (MeJA) and salicylic acid (SA) treatments. Overexpressing *AtAF2* renders plants more susceptible to *Fusarium oxysporum* [17, 18]. In general, salt stress NAC TFs are involved in regulating ethylene, growth hormone, and abscisic acid signalling pathways by binding to the promoter NACRS cis-acting element. By targeting different genes, different members of the NAC TF family mediate various pathways to regulate salt stress responses. These members tend to show differences in the sequences of neighbouring regions of the core cis-elements and bind to different target gene promoters [11]. In rice, induction of *OsNAC6* gene expression occurs under abiotic stresses such as low temperature, drought, high salt, mechanical damage, and rice blast. Overexpressing *OsNAC6* in transgenic rice enhances tolerance to water deficit and high salt stress and strengthens resistance to rice blast [19]. In *A. thaliana*, three closely related NAC proteins, namely, *AtNAC019*, *AtNAC055*, and *AtNAC072* (*RD26*), have the ability to respond to a range of abiotic stresses and hormonal treatments, including dehydration, cold, high salt, mechanical damage, jasmonic acid (JA), and abscisic acid. Expression of the rice NAC gene *OsNAC066* is induced by polyethylene glycol (PEG), H_2_O_2_, and salt treatments. Transgenic rice overexpressing *OsNAC066* exhibits increased sensitivity to abscisic acid, reduced water loss, higher proline and soluble sugar contents, and diminished accumulation of reactive oxygen species. As a result, the rice plants develop enhanced tolerance to drought and oxidative stresses [20]. Similarly, overexpressing the wheat NAC gene *TaNAC47* in *A. thaliana* amplifies sensitivity to abscisic acid, leading to a cascade of responses that alter gene expression and enhance resistance to PEG, high salt, and low-temperature stresses. Additionally, *TaNAC29*, another NAC TF in wheat, enhances salt tolerance and drought resistance and helps to regulate floral development when overexpressed in *Arabidopsis* [19].

*T. aestivum* has a moderate level of salt tolerance, surpassing that of rice but falling short of the salt tolerance shown in barley. Moreover, *T. aestivum* is regarded as a highly desirable crop that is planted in regions with high salinity levels. *Th. elongatum*, a species closely related to wheat, exhibits the ability to flourish in settings characterized by salt concentrations akin to those seen in saltwater. Octoploid *Tritipyrum*, which is derived from a hybridization event between *T. aestivum* (AABBDD) and *Th. elongatum* (EE), represents a significant germplasm reservoir for the incorporation of salt tolerance genes from *Th. elongatum* into *T. aestivum*. The comprehensive sequencing of the genomes of *T. aestivum* and *Th. elongatum* provides a solid basis for the exploration of the structure and functionality of the genes implicated in these organisms [21, 22]. The objective of this work was to investigate the genomic structural characteristics, chromosomal positions, gene duplications, and evolutionary divergence of the NAC gene family in *Tritipyrum*. Furthermore, the expression patterns of 68 *TtNAC* genes were examined under circumstances of salt stress. The acquired findings were analyzed using Pearson correlation analysis, which included the investigation of the expression levels of *TtNAC477* during both salt stress and the subsequent recovery period. The results of this study offer significant insights and recommendations for improving the salt tolerance of plants.

## Material and methods

### Plant material

*Tritipyrum* is a synthetic octoploid organism that consists of the A, B, and D genomes derived from *T. aestivum*, and the E genomes obtained from *Th. elongatum*. The stable offspring of a broad cross between *T. aestivum* and *Th. elongatum*, known as *Tritipyrum* octoploid 'Y1805', exhibits salt tolerance. The protein and nucleic acid sequences of *Tritipyrum*, which were utilized for the identification of NAC genes, were acquired from the genome databases of *T. aestivum* and *Th. elongatum*. The Phytozome 13 Plant Genomics Portal includes the genomic sequences of many plant species, namely *A. thaliana*, *Hordeum vulgare*, *O. sativa*, *Zea mays*, and *Thinopyrum intermedium*. The genomic sequences of *Secale cereal* were obtained from the China National GenBank Database (https://ngdc.cncb.ac.cn/gwh/Assembly/12832/show). To investigate transcriptome datasets available to the public, this study utilized information available at (https://www.ncbi.nlm.nih.gov/bioproject/PRJNA769794), with an accession number of PRJNA769794.

### Genomic in situ hybridization

The seeds were subjected to germination at a temperature of 25 °C, using wet filter paper as the growth medium within Petri dishes. Subsequently, the seeds were maintained at a temperature of 4 °C for an estimated duration of 24 h, after which they were relocated back to a temperature of 25 °C. Prior to being submerged in Carnoy's solution, the roots underwent a process of cutting and were thereafter immersed in cold water for a duration of around 24 h. After undergoing fixation and staining with carbol fuchsin, the mitotic chromosomes of the root ends were examined using a microscope. The DNA of *Th. elongatum* was labeled with fluorescein-12-dUTP utilizing the nick translation technique in order to function as a probe. Blocking DNA derived from Chinese Spring genomic DNA with a chromosome number of 42 was used. The transparencies were subjected to counterstaining using a solution of propidium iodide at a concentration of 0.25 mg/mL in Vectashield mounting media, purchased from Vector Laboratories in the United States [23].

### Identification of NAC genes in *Tritipyrum*

The protein sequences of NAC consensus (PF02365) were acquired from the Pfam database located at http://www.pfam.sanger.ac.uk. The identification of *TtNAC* protein candidates was conducted by employing HMMER3.0 with default settings and applying a threshold value of 0.01 for elimination. Furthermore, a total of 85 *AtNAC* sequences retrieved from the UniProt database (www.uniprot.org) were utilized for the construction of a search file library. A BLASTP search was conducted to identify *Tritipyrum* NAC (*TtNAC*) proteins by utilizing the published sequences of NAC proteins from *A. thaliana* as query sequences, specifically focusing on their NAC domain. The search criteria included a minimum score value of 100 and a maximum evalue of 1^−10^. Following the elimination of duplicate candidate sequences, the validation of the remaining candidates was conducted using the Pfam database and the SMART program [24]. The physical and chemical features of the genes were evaluated using ExPASy, a web-based tool available at http://web.ExPASy.org/protparam/.

### Phylogenetic analyses and conserved motif determination

In order to create phylogenetic trees, the amino acid sequences of NAC proteins from *A. thaliana* were obtained from the UniProt database. Additionally, a set of amino acid sequences from recently discovered NAC genes were aligned using the ClustalX 2.0 software. Phylogenetic trees of *Tritipyrum* and *A. thaliana* were created utilizing the Neighbor-Joining (NJ) technique, employing the Poisson model, and employing one thousand bootstrap replications as the particular parameters. The MEME online program (http://meme.nbcr.net/meme/intro.html) was utilized for the purpose of identifying conserved motifs throughout *TtNAC* proteins. The selected parameters for the analysis included an ideal mode width ranging from 6 to 200, and a maximum of 10 motifs. The phylogenetic tree was shown, modified, and annotated using FigTree (v1.4.4) and iTOL (https://iTOL.embl.de/).

### Chromosomal distribution, duplications and synteny analyses

The mapping methods of *TtNAC* genes to *Tritipyrum* chromosomes (combined genomes of *T. aestivum* (IWGSCv2.0) and *Th. elongatum* (v1.0)) was reported by Liu et al. [25]. The analysis of *TtNAC* gene replication events was conducted using Multiple collinear scanning toolkits MCScanX (v.1.0) using the default parameters [26]. The software DIAMOND v0.8.25 was employed with specific parameters (–max-target-seqs 5 –evalue 0.00001) to perform pairwise protein sequence comparisons required for the MCScanX analysis [27]. The classification of genes within a genome is determined by their copy number and genomic distribution. These genes can be categorized as singletons, scattered duplicates, proximal duplicates, tandem duplicates, or segmental/WGD duplicates. The Dual Synteny Plotter function in TBtools (version 1.13) [28] was employed to generate syntenic analysis graphs of the NAC gene family in *Tritipyrum*, *H. vulgare*, *O. sativa*, *S. cereal*, *Th. intermedium*, and *Z. mays*. The generated graphs were edited with Adobe Illustrator CC 2016.

### Plant growth conditions and stress treatments

The methodology employed for real-time PCR analysis was identical to that used for transcriptome data. The seeds of *Tritipyrum* octoploid 'Y1805' were subjected to germination in a controlled growth chamber with a relative humidity of 75% and a light–dark photocycle temperature of 20/15 °C. The germination process took place in 1/2 Hoagland's solution, on a floater board that maintained the same temperature and relative humidity as the germination chamber. The culture solution was replaced three times per week. Salt stress treatments were begun on the fourteenth day, namely at the two-leaf stage, using a solution consisting of 1/2 Hoagland's solution supplemented with 250 mM NaCl. Root, stem, and leaf samples of uniform size were collected from *Tritipyrum* (T1) five hours following exposure to salt stress. After 24 h of salt stress, the materials were recovered in 1/2 Hoagland's solution without NaCl. A second sample (T2) was collected one hour after the recovery time point. The parallel controls CK1 and CK2 were cultured under standard conditions (using 1/2 Hoagland's solution without NaCl). The tissue samples were expeditiously subjected to freezing in liquid nitrogen and thereafter preserved at a temperature of -80 °C for the purpose of conducting qPCR and gene cloning. A minimum of three biological replications were conducted, with each replication consisting of the combination of at least 10 seedlings.

### Expression analysis and qPCR validation of NAC genes under salt stress and recovery

The present investigation made use of transcriptome datasets accessible at the National Center for Biotechnology Information (NCBI) under the Bioproject identifier PRJNA769794, which may be found at the following URL: (https://www.ncbi.nlm.nih.gov/bioproject/PRJNA769794) [29]. The utilization of RNA-Seq datasets was applied in order to examine the expression patterns of *Tritipyrum* NAC genes during circumstances of salt stress and throughout the subsequent recovery phase. The sequencing data was subjected to preprocessing using the fastp program (https://github.com/OpenGene/fastp) with default defaults. This preprocessing step entailed the removal of reads that had adaptor contamination, low-quality bases, and uncertain bases. Subsequently, the reads of superior quality were matched to the collective genomes of *T. aestivum* (IWGSCv2.0) and *Th. elongatum* (v1.0). To ensure proper alignment, the reference genome index was generated using Bowtie version 2.2.3. Subsequently, the filtered reads were matched to the composite reference genome using HISAT version 2.2.0. The quantification of reads associated with NAC genes was performed using FeatureCounts version 1.5.1. The quantity of reads was calculated as transcripts per kilobase (TPM) [30]. Following that, the outcomes were converted and graphically shown utilizing the R Circlize package (https://CRAN.R-project.org/package=circlize). The process of Gene Ontology (GO) annotation was conducted by employing the BLAST2GO program (Biobam Bioinformatics S.L., Valencia, Spain, http://blast2go.com/b2ghome/about) and mapping GO words, with the use of an e-value threshold of 10^–6^. The process of primer design was carried out with Primer 5.0 software, as indicated in Table S2. The Actin gene (accession number: AB181991) was chosen as the internal control based on its constant expression across all growth stages and tissues. Additionally, it served as a reference for calibrating the detection of three technical repeats within each of the three biological duplicates. The 2^−ΔΔCt^ technique was utilized to ascertain the levels of expression. The real-time polymerase chain reaction (PCR) settings and the determination of relative gene expression were performed according to the methods previously outlined in reference [31].

### Data analysis

The analysis and visualization of the real-time PCR and transcriptome data were performed using statistical software (SPSS 20.0, IBM Corporation) and graphical program (Origin 2018, OriginLab). The analysis of variance (ANOVA) and Duncan's multiple range tests were employed to compare means and ascertain significant differences between means, with a predetermined significance level of *p* < 0.05. The investigation involved doing Pearson's correlation analysis on binary variables, resulting in the identification of two variables that exhibited a significant connection at a significance level of *p* < 0.05.

## Results

### Identification of *TtNAC* genes in *Tritipyrum*

**Fig. 1:**
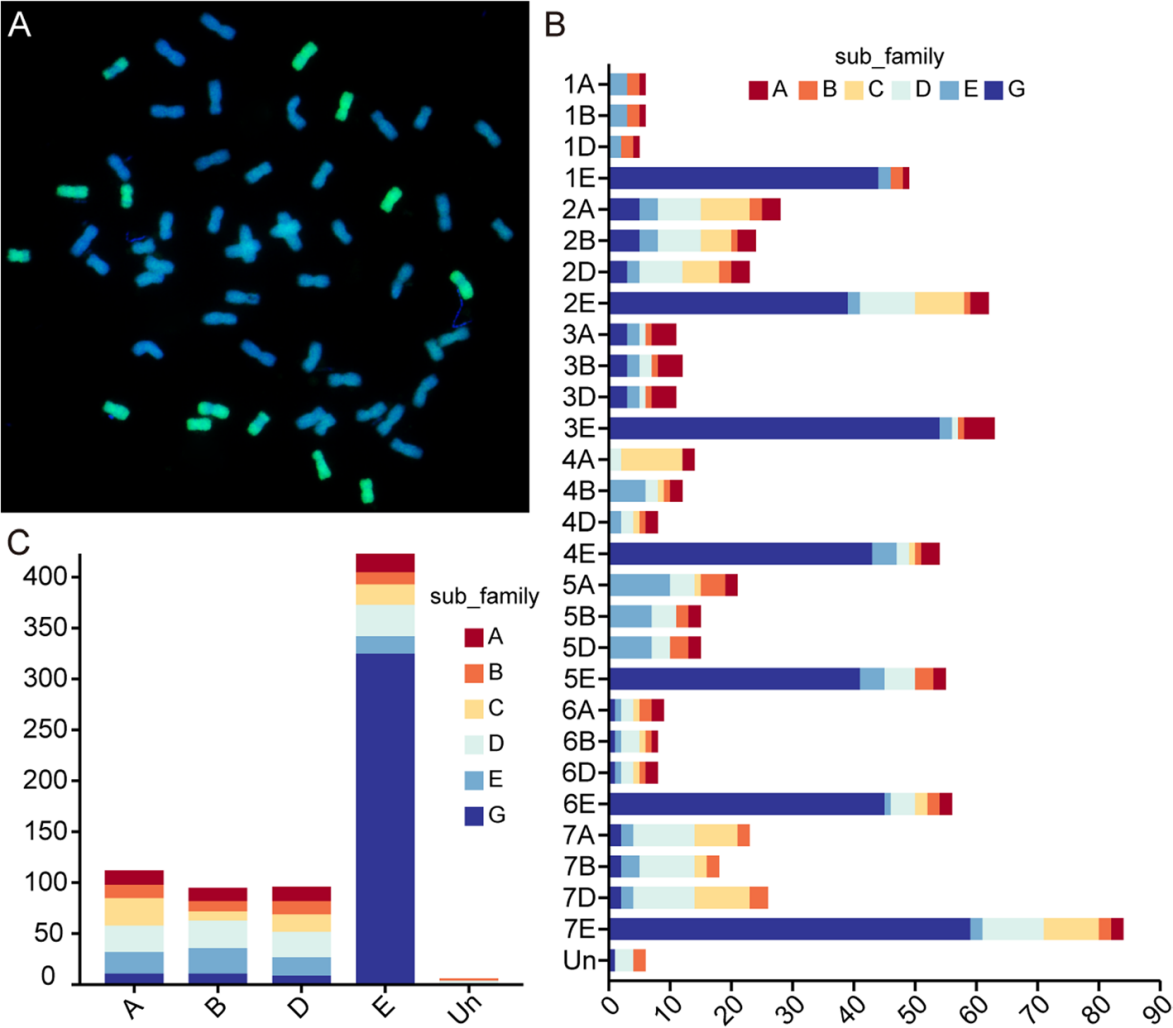
Chromosome configuration and distribution of NAC genes in *Tritipyrum*. **A **Chromosome configuration of *Tritipyrum* ‘Y1805’; **B **Subgenomes distribution of NAC genes in *Tritipyrum*; **C **Chromosomal distribution of NAC genes in *Tritipyrum*

The chromosomal count of *Tritipyrum* 'Y1805' is 56, with 42 chromosomes originating from *T. aestivum* (blue) and 14 chromosomes from *Th. elongatum* (green) (Fig. 1A). In order to ascertain the presence of NAC genes in the *Tritipyrum* genome while excluding any duplicated sequences, a combination methodology involving the utilization of hidden Markov model (HMM) and BLASTp approaches was implemented. This strategy successfully led to the identification of a total of 732 NAC genes. The NAC genes were given new names based on their precise locations on the chromosomes of *Tritipyrum*, as indicated in Supplementary Table S1. The research revealed that a significant proportion of the 732 NAC genes identified in *Tritipyrum* are situated inside the E subgenome, as depicted in Fig. 1B and C. It is noteworthy to notice that the *Tritipyrum* NAC proteins display significant variation in terms of their length and molecular weight (MW). The *TtNAC* genes are responsible for encoding proteins that exhibit a range of amino acid (aa) lengths, spanning from 120 (Tel7E01T914000.1) to 730 (TraesCS5A02G271500.2). These proteins also exhibit a comparable range of molecular weights (MWs), varying from 13.75 (Tel7E01T914000.1) to 80.1 (TraesCS5A02G271500.2). The isoelectric points (PIs) of the proteins vary between 4.12 (TraesCS1B02G277300.1) and 11.06 (Tel2E01T257700.1) (Supplementary Table S1).

### Phylogenetic analysis and motif composition of *TtNAC* genes

**Fig. 2:**
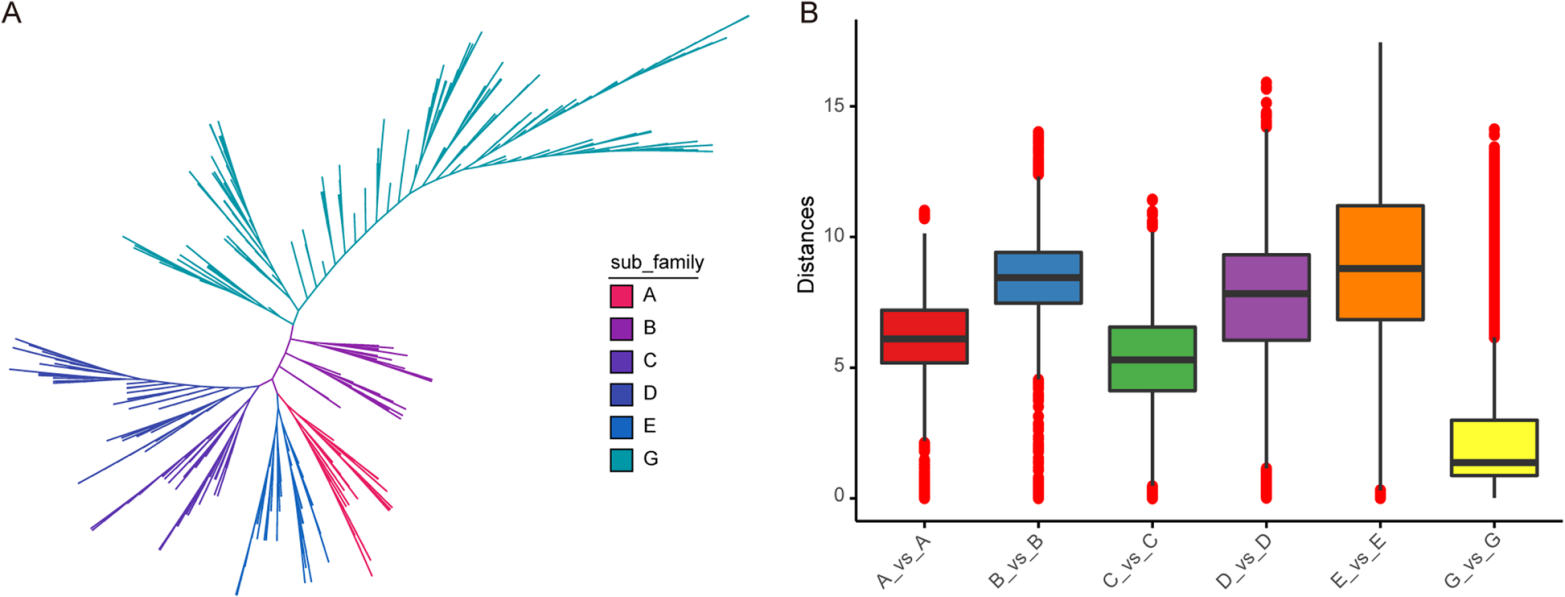
Phylogenetic relationships and distance among the NAC proteins from *Tritipyrum* and *A. thaliana*. **A **Phylogenetic relationships among 842 NAC proteins from *Tritipyrum* and *A. thaliana*; **B **genetic distance among the different clades of NAC genes. The box plot shows the median (black line), interquartile range (box), and maximum and minimum scores (whiskers) of each data set

In order to examine the evolutionary connections and categorize the NAC family in *Tritipyrum*, a phylogenetic tree was constructed with 842 potential NAC structural domains that were identified in both *A. thaliana* and *Tritipyrum* (as shown in Fig. 2A and Fig. S1). The *Tritipyrum* NAC family was classified into six main groups, excluding subgroups F and H, based on the classification of *AtNAC* and the primary amino acid sequence properties of the model organism *A. thaliana*. Within the aforementioned groups, it is evident that Group G has the highest magnitude in terms of subfamily size, encompassing a total of 357 proteins. Conversely, Group B is characterized by its very diminutive nature, comprising a mere 50 proteins. Moreover, it is seen that the *TtNAC* proteins exhibit a higher degree of similarity to *Arabidopsis* NAC proteins compared to *Tritipyrum* NAC proteins. Additionally, a significant proportion of *TtNAC* proteins belonging to the same group demonstrate consensus motifs, as depicted in Fig. S1. It is worth mentioning that the C and G subfamily factors, namely the G subgroup, exhibit significant conservation across *Tritipyrum* and *A. thaliana*, as depicted in Fig. 2B. The degree of variation observed between subgroups D and E is comparatively higher than the variation observed among the remaining subgroups. Furthermore, it is worth noting that *Tritipyrum* and *A. thaliana* have noteworthy genetic divergence specifically between subgroup G and the remaining subgroups, as illustrated in Fig. 2B and Supplementary Fig. S2.

### Chromosomal distribution, gene duplication and synteny analysis of *TtNAC* genes

**Fig. 3:**
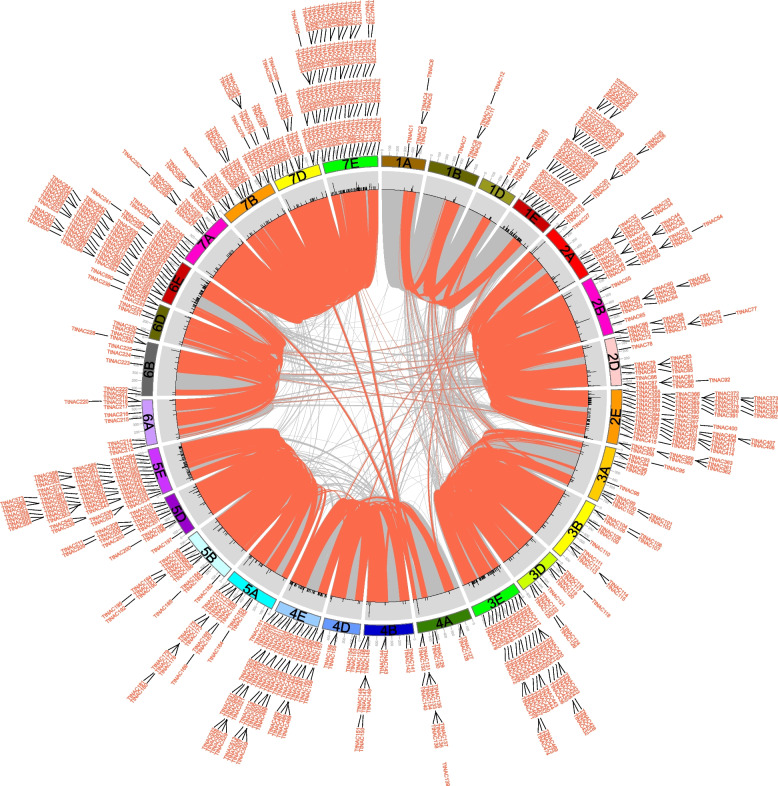
Distribution, duplication and synteny analysis of NAC genes in *Tritipyrum*. Collinear correlations of NAC in *Tritipyrum* genomes are displayed by Circos. *Tritipyrum* chromosomes are colored according to the inferred ancestral chromosomes following an established convention. In the center, the relative map position of 726 NAC genes is shown on each of the 28 *Tritipyrum* chromosomes

The chromosomal positions of the *TtNAC* genes were examined, revealing that out of the total of 732 genes, 726 were found to be distributed throughout 28 chromosomes. The six remaining *TtNAC* genes were unable to be assigned to a specific chromosome due to their placement on scaffolds (Supplementary Table S1, Fig. 3). The second (18.7%) and seventh (20.6%) homologous clusters contained the highest proportion of *TtNAC* genes, while the first (9.1%) and sixth (11.1%) homologous groups had a smaller number of these genes (Supplementary Table S1, Figs. 1 and 3). The majority of *TtNAC* genes are positioned towards the telomeres in terms of their chromosomal location, while only a limited number are found closer to the centromeres (see Fig. 3). Furthermore, a comprehensive examination of gene duplication events has demonstrated that the *TtNAC* genes experienced both proximal and tandem duplications. A total of 34 pairs of genes that were duplicated in close proximity and 76 pairs of genes that were duplicated in tandem were discovered in our study (Fig. 3). The distribution of homologous genes primarily occurs within the same homologous groups, with a limited presence in the fourth, fifth, and seventh homologous groups. This pattern is consistent with the natural translocations that took place during the developmental and evolutionary processes of *T. aestivum*.

### Evolutionary analysis of NAC families in several different species

**Fig. 4:**
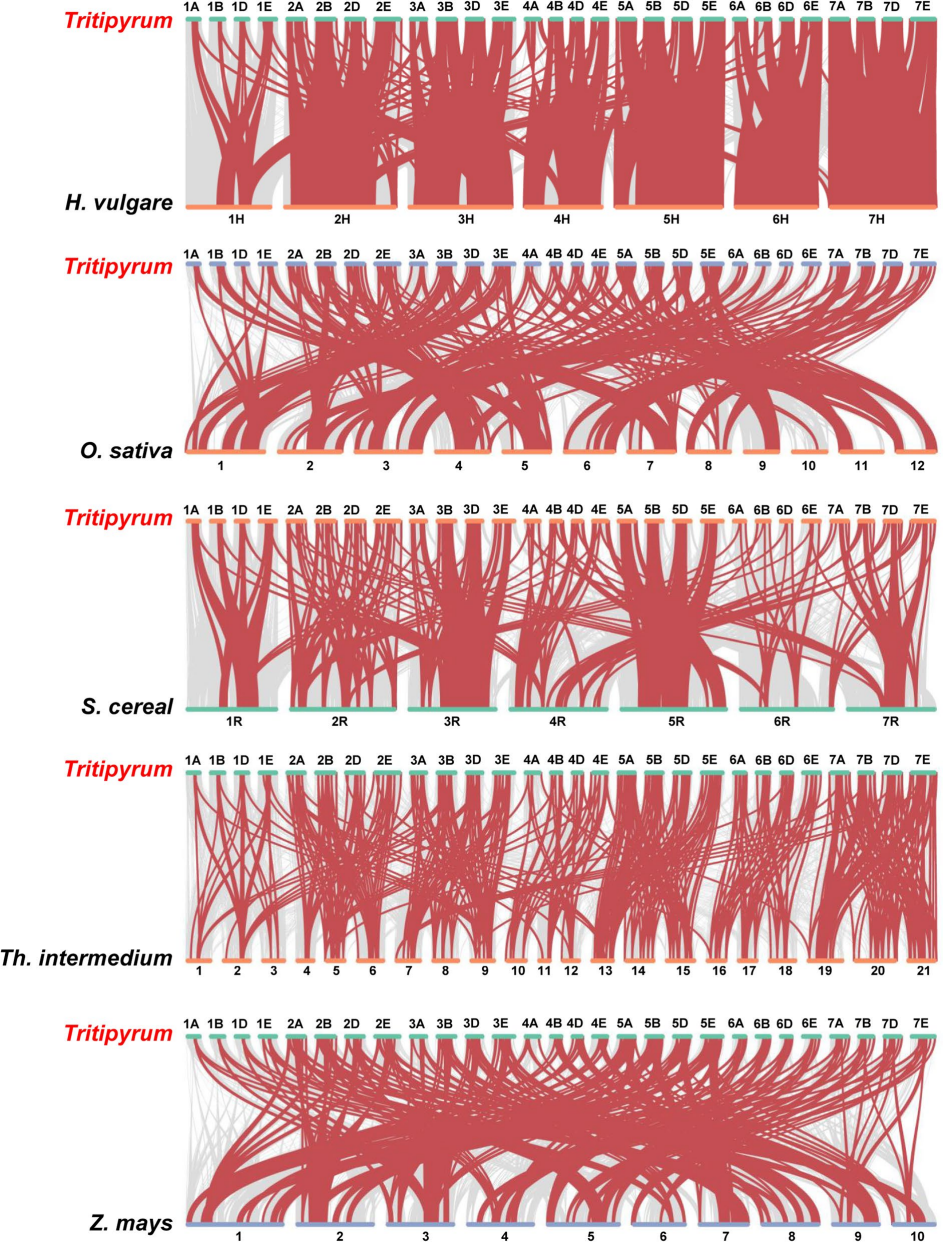
Synteny analyses between *Tritipyrum* and five representative plant species. Gray lines in the background indicate collinear blocks within *Tritipyrum* and other plant genomes, while red lines highlight syntenic NAC gene pairs

In order to gain a deeper understanding of the evolutionary links within the NAC gene family, we conducted an analysis of the syntenic relationships across six species: *Tritipyrum*, *H. vulgare*, *O. sativa*, *S. cereal*, *Th. intermedium*, and *Z. mays*. Figure 4 depicts five discrete categorizations of syntenic links. The highest frequency of syntenic linkages was observed between *Tritipyrum* and *Th. intermedium*, with 254 *TtNAC* genes. This was followed by *Z. mays* with 139 genes, *O. sativa* with 118 genes, *H. vulgare* with 115 genes, and *S. cereal* with 104 genes (Fig. 4). Collinear couples were identified between *Tritipyrum* and the remaining five species, suggesting the presence of orthologous pairs predating the original divergence. Furthermore, it was shown that certain NAC collinear gene pairs, which were discovered in *Tritipyrum* and *H. vulgare*, were associated with extensively conserved syntenic blocks including more than 500 collinear sites. Both *Tritipyrum* and *O. sativa* have comparable patterns. Several *TtNAC* genes exhibit connections with a minimum of three syntenic gene pairs, indicating that these genes may have played a pivotal role in the evolutionary development of the NAC gene family. The results of this study provide evidence for the significant conservation of the *TtNAC* gene family and the closer phylogenetic link between the *TtNAC* genes in *Z. mays* and *S. cereal.*

### Expression of *TtNAC* genes under salt stresses and recovery

**Fig. 5:**
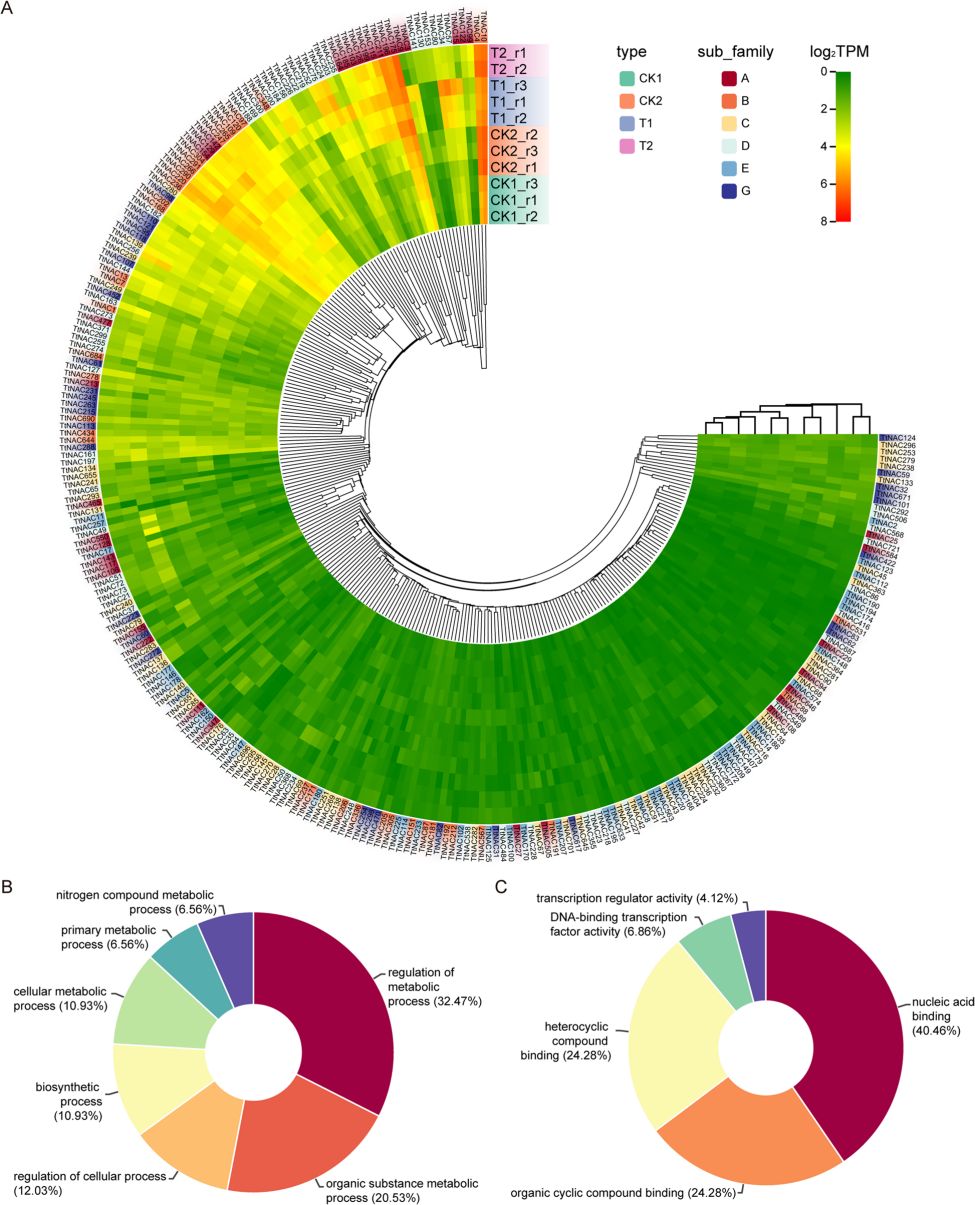
Expression patterns of *TtNAC* genes under salt stresses and recovery treatments. **A **Hierachical clustering of expression profiles of 266 *TtNAC* genes were expressed in 11 samples including salt stress and recovery treatment. **B** and **C **the BP (**B**) and MF (**C**) analysis of 266 expression genes

In order to examine the effects of various salt stresses and recovery treatments on the expression of *TtNAC* genes, a thorough investigation was undertaken on the transcriptional levels of all 732 *TtNAC* genes. Out of the 11 samples that were subjected to testing, it was observed that a total of 266 *TtNAC* genes exhibited consistent expression across all samples. Additionally, 106 genes had constitutive expression, as indicated by a transcript per million (TPM) value greater than 1 in each of the samples. The results of the cluster analysis indicated that there was no significant association between the subfamily types of the 732 *TtNAC* genes and the salt stress and recovery treatments (Fig. 5A). Furthermore, it was observed that 446 *TtNAC* genes exhibited no expression across all samples, indicating the possibility of their classification as pseudogenes or their lack of participation in salt stress and recovery mechanisms. Additional analysis of the 266 genes that were identified revealed their involvement in diverse biological processes (BP) and molecular functions (MF). These include the regulation of metabolic processes, metabolism of organic substances, cellular processes, biosynthetic processes, cellular metabolism, nucleic acid binding, binding to organic cyclic compounds, binding to heterocyclic compounds, DNA-binding transcription factor activity, and transcription regulator activity (Fig. 5B and C).

**Fig. 6:**
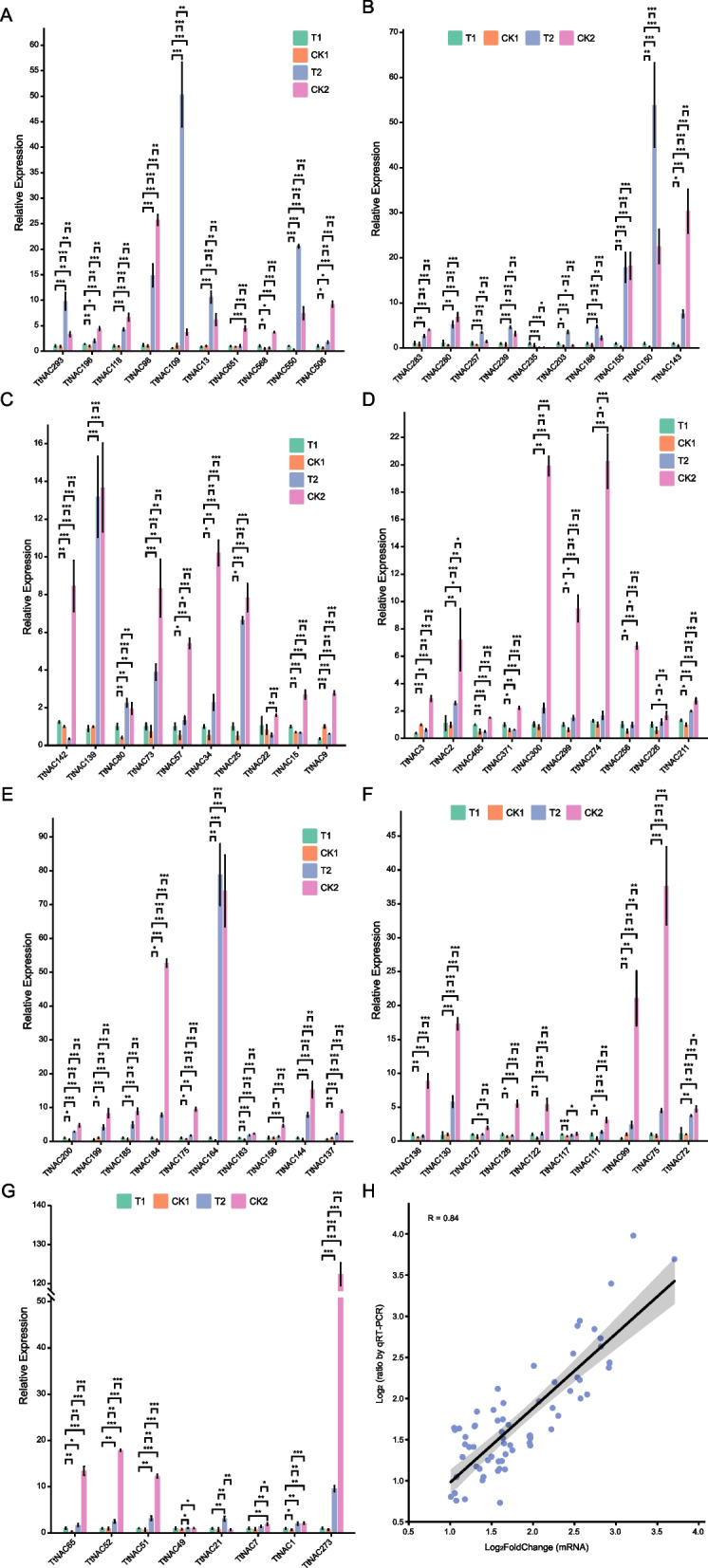
**A**-**G**: Expression analysis of 68 NAC genes in eleven samples by qPCR. Data were normalized to β-actin gene and vertical bars indicate standard deviation. **H** The relationships between qPCR and transcriptional of 68 up-regulated expression genes. Values are the log_2_ratio (salt stress or recovery treatment /CK treatment) for genes. The determine coefficient (r^2^) is indicated in the Figure. All qPCR reactions were performed in three biological replicates

In order to verify the reliability of the transcriptome data, a subset of 68 *TtNAC* genes was chosen from a larger set of 266 *Tritipyrum* NAC genes. These 68 genes were selected based on their relatively high mRNA levels (log_2_FoldChange > 1) in response to different salt stress and recovery protocols. Specific primers were then designed for each of these 68 gene loci (Supplementary Table S2). In addition, we performed quantitative real-time PCR (qPCR) investigations to evaluate the expression patterns of these genes in relation to various salt stress and recovery therapies. The quantitative polymerase chain reaction (qPCR) results demonstrated similar variations to the transcriptome data for the specific genes analyzed (Fig. 6A-G). In addition, it is worth noting that the gene expression patterns exhibited a significant degree of resemblance (*r*^2^ = 0.84) to those observed in the transcriptome data, so providing further validation for the accuracy and dependability of our transcriptome findings (Fig. 6H).

### Expression patterns and correlation analysis of *TtNAC477*

**Fig. 7:**
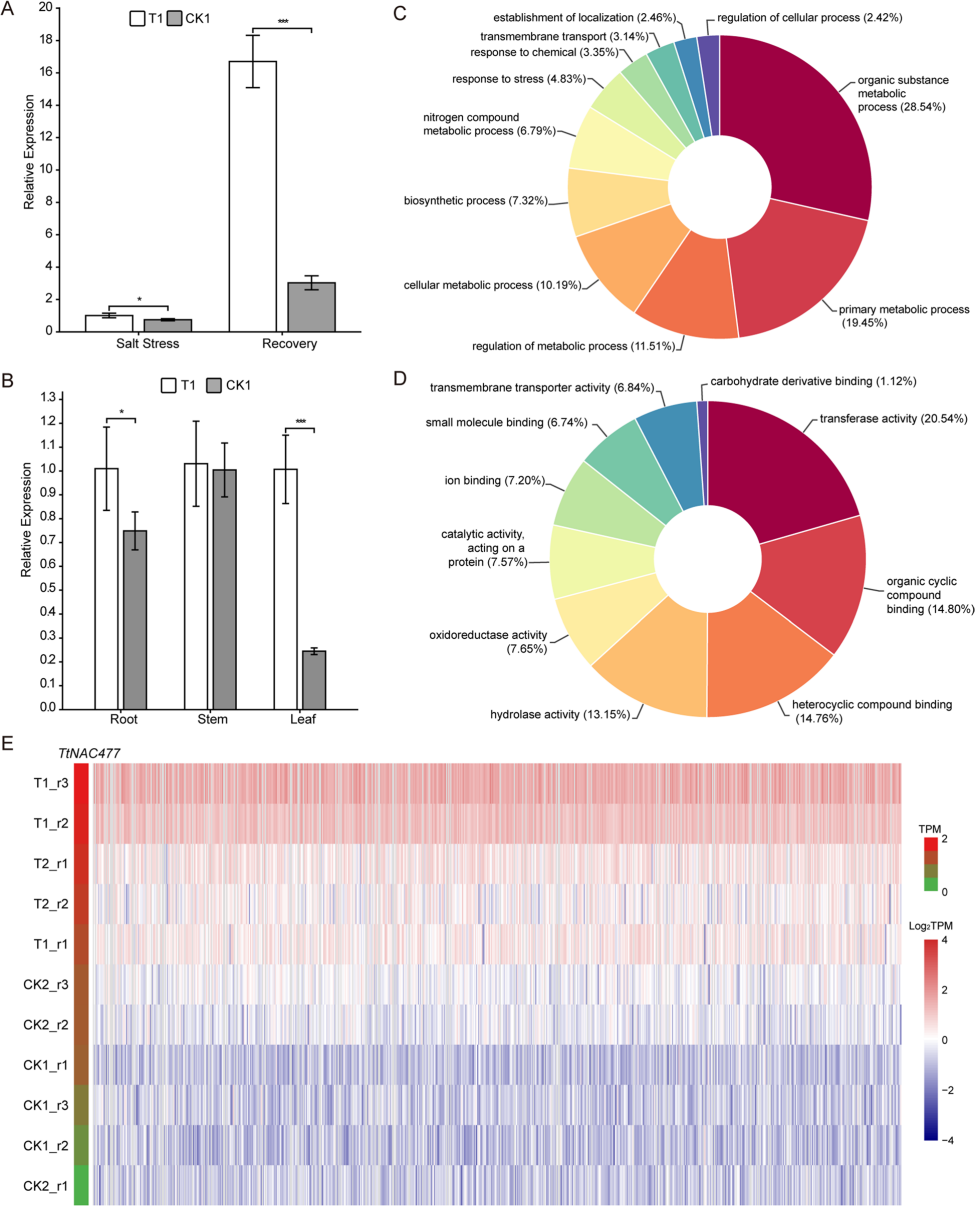
Expression patterns and correlation of *TtNAC477*. **A **Relative expression levels of *TtNAC477* in roots under salt stress and recovery conditions; **B **Relative expression levels of *TtNAC477* in roots, stems, and leaves under salt stress; **C** and **D **the BP (**C**) and MF (**D**) analysis of 751 positively related to *TtNAC477* expression genes; E: seven hundred fifty-one genes positively related (*R* > 0.9) with *TtNAC477* expression

Previous research has indicated that two proteins belonging to the *A. thaliana* NAC family, specifically *ANAC019* (AT1G52890) and *ANAC055* (AT3G15500), have transcriptional activation properties and are involved in the regulation of defense gene expression triggered by JA. Moreover, it has been observed that the gene *TtNAC477* (Tel3E01T644900) belonging to the *Tritipyrum* NAC gene family shares an evolutionary lineage with *ANAC019* and *ANAC055* (as depicted in Fig. S1). Additionally, it has been found that the expression of *TtNAC477* is enhanced under different salt stress conditions and during the subsequent recovery phase (as illustrated in Fig. 5). In order to investigate the spatial and temporal distribution of *TtNAC477* expression, quantitative polymerase chain reaction (qPCR) was employed to quantify its transcript levels in various root tissues subjected to salt stress and subsequent recovery conditions. The expression levels of *TtNAC477* were found to be significantly elevated in the roots subjected to both salt stress (1.34-fold) and recovery (5.51-fold) treatments, in comparison to the control group (Fig. 7A). The findings are consistent with the transcriptome data that was previously provided. Furthermore, it was observed that the expression level of *TtNAC477* was significantly elevated in the stems of 'Y1805' under salt stress conditions, with the highest relative expression level. This was followed by the leaves and subsequently the roots, as depicted in Fig. 7B.

A Pearson correlation analysis was performed to examine the biological processes linked to the expression of *TtNAC477* during conditions of salt stress and subsequent recovery. The analysis encompassed *TtNAC477* as well as other genes present in the transcriptome data. The findings of the study indicated that a total of 751 genes exhibited a statistically significant positive correlation (*R* > 0.9) with the expression of *TtNAC477*. The expression levels of these genes were found to be significantly elevated in samples T1 and T2, as depicted in Fig. 7E. The genes were subjected to Gene Ontology (GO) analyses, which revealed significant biological process (BP) and molecular function (MF) terms. These terms encompassed organic substance metabolic process, primary metabolic process, regulation of metabolic process, cellular metabolic process, and biosynthetic process (Fig. 7C and D). In summary, the aforementioned findings indicate a plausible association between *TtNAC477* and the capacity of plants to withstand abiotic stress.

## Discussion

NAC proteins, a family of plant TFs, have been extensively studied in various plant species to understand their evolution, function, and response to abiotic adversity. In this study, we identified 732 members of the NAC gene family in *Tritipyrum*. The distribution of conserved amino acids within the NAC structural domain of *Tritipyrum* is highly similar to that of *Arabidopsis*, suggesting conservation of NAC during plant evolution (Fig. S1). Specific motifs within a gene family may be associated with distinct plant biological processes, and genes with identical motifs within the same subfamily are likely to have similar functions. Therefore, each NAC gene family or subfamily may be linked to specific biological processes [32]. Based on functional classification of *TtNAC* using the *Arabidopsis* model, we examined the shared motifs and potential functions of each *TtNAC* group in greater detail. Furthermore, our analysis revealed that NAC TFs are unevenly distributed across all chromosomes, with the E subgenome having the highest abundance of NACs and the B subgenome having relatively few NACs. Similar uneven chromosome distributions of NAC genes have been observed in rice [32] and maize [34]. Additionally, gene clustering is commonly observed in the distribution of NAC family genes in rice, poplar, and soybean [33, 35, 36]. The three primary types of gene duplications include genome-wide duplications, tandem duplications, and segmental duplications [37]. Most plants have undergone genome-wide or segmental duplications [38], which involve large-scale doubling of segments of chromosomes retained in the genome. Duplicated genes may exhibit variations in expression. These evolutionary processes, such as duplication and polyploidization, contribute to expansion of gene families in plants [39, 40]. Moreover, point mutations occurring in coding DNA regions and regulatory sites of duplicated genes can alter the function of the newly formed copies [41, 42]. Our analysis identified 34 pairs of nearly identical duplicated genes and 76 pairs of tandem duplicated genes in *Tritipyrum* (Fig. 3). These findings suggest multiple copies of some genes in the *T. aestivum* genome, likely due to multiple duplications during replication. We further investigated the covariance between the NAC genomes of *Tritipyrum*, *H. vulgare*, *O. sativa*, *S. cereal*, *Th. intermedium*, and *Z. mays*. The highest covariance linkages were observed between *Tritipyrum* and *Th. intermedium*. This information supports a close evolutionary relationship between these taxa, consistent with the traditional classification of graminoids, with *TtNAC* genes from multiple plants possibly originating from a common ancestor. In contrast, the two distinct genera, *Tritipyrum* and *S. cereal*, exhibit the fewest covariance linkages, likely due to their separate evolutionary paths. Notably, at least three sets of collinear relationships share 3–21 NAC genes, providing insights into the evolution of NAC genes across different species.

Salinity poses a significant environmental threat to crop production due to its detrimental effects on plant performance and disruption of cellular metabolism. The adverse impact of increased salinity is primarily attributed to osmotic stress and accumulation of harmful ions within plant cells [43]. Unlike single functional genes, TFs have the ability to regulate multiple downstream salt stress response genes simultaneously. Overexpression of TFs related to salt tolerance in plants has been demonstrated to enhance their tolerance to this stress. Plant genes encoding TFs are differentially expressed as part of a complex stress response mechanism. Notably, bHLH, bZIP, MYC, WRKY, and NAC proteins are among the TFs associated with the salt tolerance pathway [44–46]. The corresponding NAC genes in response to various environmental stresses have been identified in *Arabidopsis*, rice, maize, and wheat [20, 47–50]. Tissue-specific expression of NAC genes, through regulation of transcriptional processes, can influence the growth and development of target tissues and organs [11, 19]. For instance, Mao et al. investigated the *TaNAC67* TF in wheat using transgenic *A. thaliana*. Their study demonstrated that overexpression of *TaNAC67* in transgenic *Arabidopsis* plants significantly improved tolerance to drought, high salt, and cold stresses without adversely affecting normal growth [51]. Similarly, in rice, overexpression of endogenous genes *SNAC1*, *OsNAC6*, *SNAC2*, *ONAC045*, *OsNAP*, *ONAC106*, *ONAC022*, and *OSNAC2* [50, 52–55] or exogenous genes such as *ATAF1* and *EcNAC67* led to varying degrees of tolerance to cold, drought, and salt stresses, either alone or in combination [18, 56]. Similarly, heterologous overexpression of NAC members from different species in *A. thaliana* exhibited similar results [47, 49, 57–62]. In *Arabidopsis*, ectopic expression of the pumpkin *CmNAC1* gene promoter led to an ABA-hypersensitive response and enhanced tolerance to salt stress, drought stress, and low-temperature stress [62]. Most NACs positively regulate stress responses, and only a few have been found to exhibit negative regulation. For instance, *A. thaliana ANAC069* and maize *ZmNAC071* can negatively regulate high salt and osmotic stresses by decreasing reactive oxygen species (ROS)-scavenging capacity and proline content [48, 63]. In this study, we detected 266 *TtNAC* genes in the root tissues of triploid wheat that were significantly responsive to salt stress induction. Among them, 106 *TtNAC* genes exhibited intrinsic expression and may be involved in regulating essential cellular processes. Further analysis focused on 68 of these *TtNAC* genes revealed their potential positive role in the salt stress response in triploid wheat root tissues, as indicated by expression profiling and qPCR analysis. Transgenic experiments will be conducted to further understand the precise biological functions of these *TtNAC*s and explore their application in genetic engineering for enhancing crop stress tolerance and other agronomic traits.

*AtNAC019*, *AtNAC055*, and *AtNAC072* (*RD26*) exhibit responsiveness to a wide range of abiotic stresses and hormonal treatments in *Arabidopsis*, including dehydration, low temperature, high salt, mechanical damage, jasmonic acid (JA), and abscisic acid [19]. Overexpression of *AtNAC072*/*RD26* in *Arabidopsis* results in increased expression of ABA-induced and stress-related genes, heightened sensitivity to ABA, and improved fruit resistance. Conversely, expression of these genes is suppressed in *AtNAC072*/*RD26*-repressed and ABA-insensitive plants, indicating the significant role of *AtNAC072*/*RD26* in the stress response and ABA signalling pathway [64]. Following ABA treatment, expression of key genes of the ABA pathway (*RD29A*, *RAB18*, and *RD29B*) is markedly upregulated in wild-type and NAC mutant plants. However, in the NAC triple mutant *nac019nac055nac072* and NAC double mutant *nac019nac072* and *nac019nac055*, induced expression of *RD29A*, *RAB18*, and *RD29B* did not significantly differ from that of wild-type. Nevertheless, both the single mutant *nac072* and double mutant *nac055nac072* show significant upregulation, with the latter exhibiting the most significant change in expression. This suggests a potential negative regulatory role for *AtNAC072* and *AtNAC055* in downstream gene regulation in response to ABA, and *AtNAC019* may act as an antagonist of the other two. The *TtNAC477*, *AtNAC019*, *AtNAC055*, and *AtNAC072* genes, belonging to the NAC gene family of both *Arabidopsis* and *Tritipyrum*, are located on the same evolutionary branch. Expression of *TtNAC477* was upregulated in various salt stress and recovery treatments, making it a suitable target for further functional studies. Relative expression levels of the *TtNAC477* gene in the leaves, stems, and roots of ‘Y1805’ were significantly higher than those in the control. The high salt solution inflicted direct and severe damage to the root system. Overall, expression levels of *TtNAC477* in whole plants were significantly elevated during salt stress and recovery compared to the control, which aligns with our transcriptomic data and previous findings. There is evidence that the interplay among ion homeostasis, mineral nutrition, and growth response during salt stress leads to regulated, conserved, and differentiated changes in primary and secondary metabolism within a plant. These changes trigger substantial modifications in metabolic phenotypes as a response to salt adaptation [65]. In this study, a total of 751 genes were found to have a strong positive correlation (*R* > 0.9) with expression of *TtNAC477*. Remarkably, these genes exhibited elevated expression in the T1 and T2 samples, particularly in relation to metabolism and biosynthesis according to GO analysis. *TtNAC477* was significantly and sensitively expressed throughout the plant, contributing to its ability to endure salt stress.

The problem of soil salinity poses a considerable obstacle to the processes of seed germination, seedling development, and crop output. *Tritipyrum*, an interspecific hybrid derived from the cross between *T. aestivum* and *Th. elongatum*, assumes a pivotal function in the integration of genes associated with salt tolerance into wheat [66, 67]. The enhancement of plant salt tolerance generally revolves on the activation of stress-responsive genes, which in turn generate proteins that aid in the restoration of salt-induced primary and secondary stress. Transcription factors (TFs) possess the unique capability to control a cluster of downstream target genes, hence influencing several physiological and biochemical processes in reaction to salt stress [43, 68]. This work encompasses a thorough examination of the NAC family in *Tritipyrum* through the application of bioinformatics methodologies, including as phylogenetic, motif, and correlation studies. In our investigation of *Tritipyrum*'s transcriptome response to salt stress, we employed RNA-Seq technology to discern the involvement of NAC transcription factors (TFs) in the plant's reaction to salt stress. In this study, we utilized the *Arabidopsis* salt stress-related genes *AtNAC019*, *AtNAC055*, and *AtNAC072* as reference genes to identify the *TtNAC477* gene. Furthermore, we investigated the expression levels of *TtNAC477* under both salt stress and the subsequent recovery period. Given the complex nature of plants' response to salt stress, it is imperative to ascertain the potential interactions between transcription factors (TFs), as well as their interactions with other proteins and downstream target genes. Additional investigation is required to comprehensively comprehend the potential and role of the *TtNAC* transcription factors (TFs) in salt tolerance, as their functional characteristics have not been assessed in a transgenic context. The production of salt-tolerant crops may be facilitated via the identification and application of TF candidate genes related with salt tolerance on a large scale, as well as the continuous improvement of our comprehension about the underlying processes of salt tolerance. This can be achieved through the utilization of genetic engineering techniques.

## Conclusions

The *Tritipyrum* NAC gene family was examined comprehensively in this investigation. The motif compositions of 732 full-length NAC genes show remarkable similarity within the same groups and subgroups. Phylogenetic and synteny analyses were conducted on the NAC genes from multiple plant species to shed light on their evolutionary characteristics in *Tritipyrum*. Based on their expression patterns in various tissues and their response to salt stress and recovery interventions, a total of sixty-eight *TtNAC* genes were found to play a crucial role in *Tritipyrum*'s salt stress response. In addition, *TtNAC477* has the potential to serve as a target gene for enhancing wheat salinity tolerance through biotechnology or molecular breeding. These findings provide valuable insights into the biological functions of specific NAC genes in *Tritipyrum*.

### Supplementary Information

**Additional file 1: Supplementary Table S1.** List of the TtNAC genes identified in this study.

**Additional file 2: Supplementary Table S2.** Primers used in qRT-RCR experiments.

**Additional file 3: Fig.s1.** Phylogenetic trees of NAC gene family in *Tritipyrum* and *A. thaliana.*

**Additional file 4: Fig. S2.** Genetic distance among the NAC genes in different clades.

## Data Availability

The datasets analysed during the current study are available in the National Center for Biotechnology Information repository, [https://www.ncbi.nlm.nih.gov/bioproject/PRJNA769794, accession number- PRJNA769794].
