# Supplementary figures and images for: Genome-wide analysis of the Tritipyrum NAC gene family and the response of TtNAC477 in salt tolerance
Source: BMC Plant Biol. 2024 Jan 9;24:40. doi: 10.1186/s12870-023-04629-6 (PMC10775630; doi:10.1186/s12870-023-04629-6)

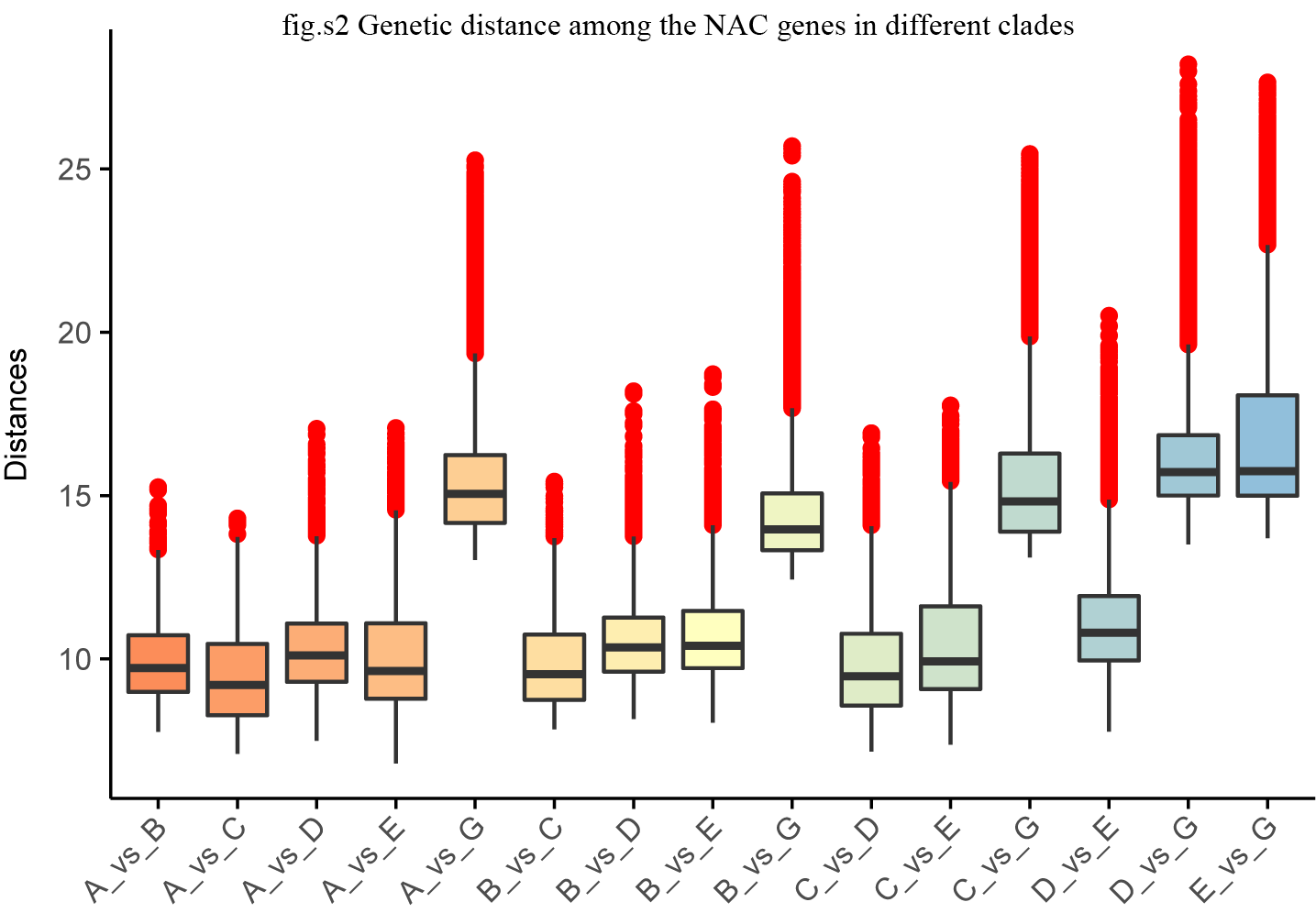

Supplement: Supplementary file 4 — Additional file 4: Fig. S2. Genetic distance among the NAC genes in different clades. [file 12870_2023_4629_MOESM4_ESM.tif]
