# Supplementary material for: Genome-wide analysis of the Tritipyrum NAC gene family and the response of TtNAC477 in salt tolerance
Source: BMC Plant Biol. 2024 Jan 9;24:40. doi: 10.1186/s12870-023-04629-6 (PMC10775630; doi:10.1186/s12870-023-04629-6)

fig.s1 Phylogenetic trees of NAC gene family in *Tritipyrum* and *A. thaliana*

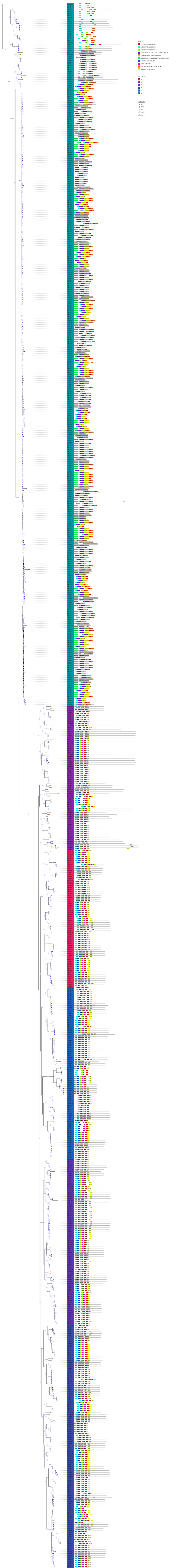

Supplement: Supplementary file 3 — Additional file 3: Fig.s1. Phylogenetic trees of NAC gene family in Tritipyrum and A. thaliana. [file 12870_2023_4629_MOESM3_ESM.pdf]
